# Establishment of Economic Forecasting Model of High-tech Industry Based on Genetic Optimization Neural Network

**DOI:** 10.1155/2022/2128370

**Published:** 2022-04-30

**Authors:** Kai Gao, Tingting Liu, Bin Hu, Miao Hao, Yueran Zhang

**Affiliations:** ^1^School of Management, Shanghai University of Engineering Science, Shanghai 201620, China; ^2^School of International Trade and Economics, Shanghai Lixin University of Accounting and Finance, Shanghai 200030, China

## Abstract

Scientific and accurate prediction of high-tech industries is of great practical significance for government departments to grasp the future economic operation and formulate development strategies. In this paper, aiming at some shortcomings of neural network (NN) applied in economic forecasting, GANN was introduced to construct the economic forecasting model of high-tech industry. Genetic algorithm (GA) has simple calculation and strong robustness and can generally ensure convergence to the global optimum, which effectively overcomes the shortcomings of NN using gradient descent method. In order to verify the feasibility of the economic forecasting model in this paper, the comparative experiments of different models are carried out in this paper. Experimental results show that the proposed algorithm has faster convergence speed and greater generalization ability, and the average error rate is reduced to about 1%. The prediction accuracy of this model reached 95.14%, which was about 11.93% higher than the previous model. Applying the economic forecasting model in this paper to the economic forecasting of high-tech industries can provide the means and reference value for the government to formulate regional future economic development plans, forecast, and control the economic growth and development direction.

## 1. Introduction

With the rapid development of information technology, the pace of economic development is also accelerating [1]. The rise and growth of high-tech industry has become an important industry to promote economic growth in today's world, which has a great influence on the position and role of a country or region in the world pattern in economic, political, military, and other aspects [2]. In the era of the “knowledge economy,” high-tech industry is at the heart of all industries, the most important motivator for a country or region to gain a long-term competitive advantage, and the engine of social economy development. The advancement of high-tech industry is not only an important tool for optimizing and upgrading industrial structure but it is also a critical factor in achieving economic multiplication [3]. As a result, in countries and regions all over the world, the development of high-tech industries has become a strategic focus of economic and social development. One of the topics that the Chinese government and economists are paying increasing attention to is how to accurately and reasonably predict the industrial economic situation based on relevant industrial economic indicators [4]. Prediction is a study that compares the results of things that will happen with reality, and it is an estimate and speculation of things that have not yet happened and are not clear at this time. Economic forecasting, as an important branch of forecasting, has gotten a lot of attention in the economic field [5]. For government departments to grasp the future economic operation and formulate development strategies, scientific and accurate predictions of high-tech industries are critical.

The economic forecast of high-tech industry plays a vital guiding role in implementing effective economic macrocontrol by national departments at all levels and making specific production and delivery by various enterprises under the market economy. Although traditional economic forecasting and early warning methods have their own advantages, with the complication of economic management problems, these traditional methods are increasingly unsuitable for scientific management and precise management. Therefore, it is particularly important to find a more general and accurate economic forecasting model. Neural network (NN) [6–8] is an important Artificial intelligence (AI) technology [9–11]. NN is also one of the fastest-growing research achievements in AI field recently. It has been successfully used in scientific calculation and automatic control. In recent years, domestic and foreign scholars have carried out a lot of applied research on NN in economic fields such as economic prosperity analysis, economic time-series prediction, portfolio optimization and stock prediction, and achieved good application results. The most widely used NN in the economic field is BPNN (back-propagation neural network). As it is essentially a gradient descent method, and the objective function to be minimized is complex, it has some shortcomings. For example: (1) The learning process converges slowly. (2) It is easy to fall into local minima and the algorithm is incomplete. (3) Poor robustness and poor network performance. Therefore, this paper introduces genetic algorithm (GA). GA is a computational model developed in recent years, which opens up a new way to solve complex problems. GA is an algorithm model to simulate the evolution process or evolution process in nature. It simulates the evolution process of species from low level to high level, adopts the natural law of survival of the fittest to select individuals, generates the next-generation population through mating and mutation and evolves from generation to generation until individuals who meet the conditions are produced [12]. GA is based on strict theory. It has strong adaptability and robustness and is especially suitable for searching for optimal solutions in high-dimensional, multipole, nondifferentiable, continuous, or discrete space. In this paper, based on genetic algorithm neural network (GANN), the economic prediction model of high-tech industry is constructed, and its innovations are as follows:

1. In this paper, GANN is introduced to overcome some shortcomings of NN in economic forecasting. Combining the two methods, GA, with its unique characteristics, effectively overcomes the shortcomings brought by NN using gradient-descent method. In this paper, GANN is applied to the economic forecast of high-tech industries and good results are obtained.
2. In view of the large scale of modern industrial economy and the uncertainties such as nonlinearity and time-varying brought about by globalization, this paper puts forward the research assumption of intelligent modeling and prediction. Combining structural self-organizing genetic optimization NN with reinforcement learning theory and method, the economic prediction model of high-tech industry is studied. The growth rate data and time-window series data of economic indicators are introduced into the constructed NN input layer unit, which improves the generalization ability of the established NN model.

The research content and structure of this paper are mainly divided into five chapters:

The introduction is the first chapter. This section introduces the paper's research background, significance, contents, and methods. The second chapter primarily summarizes and examines related literature from both within and outside the United States. Based on these documents, the second chapter introduces the innovation and research methods in general. The third chapter focuses on related theoretical high-tech industry development research and the development of a genetic optimization economic forecasting model.  Section 3.1, for example, examines the concept connotation of high-tech industry and summarizes the basic theories relating to its development. In addition, the contents of artificial neural network (ANN) and GA were introduced. The construction method and implementation of the GANN model are discussed in Section 3.2. The experimental analysis section is covered in the fourth chapter. The actual test is carried out in this section, and the test results are obtained, based on the relevant data. The model's performance is evaluated in comparison to the experimental results of other models. The fifth chapter concludes with a prognosis. This section primarily reviews the main contents and findings of economic forecasting model research, summarizes the findings, and suggests future research directions.

## 2. Related Work

The scope of China's high-tech industries and their products was determined by Zeng et al., who also analyzed the characteristics of high-tech industries, as well as the conditions and methods of developing high-tech industries and outlined the basic theories related to high-tech industry development [13]. Kuo et al. proposed a construction scheme for a stock price forecasting system based on the vector error correction model [14] based on previous research in the field of economic forecasting and early warning. Liu et al. proposed a rolling grey model economic forecasting algorithm based on particle swarm optimization [15] to address the uncertain subjective factors in economic forecasting and control. To predict the industrial economy, Yuan et al. developed a GA-based BPNN model and compared it to a regression analysis model [16]. Claveria et al. used special processing methods to create a general economic forecasting model by combining the characteristics of economic time series. And then use this model to forecast economic data [17]. For the control and prediction of regional macroeconomics, Rossi et al. developed a prediction model based on the intelligent NN algorithm. Modeling and predictive control methods are proposed to be improved [18]. The NN model is used for multivariate time-series forecasting, according to Zhao et al., and its accuracy and trend are better than traditional statistical methods. The experimental results show that the method is feasible and that it has a higher prediction accuracy than one-shot modeling [19]. The use of NN for multivariate time-series forecasting and multivalue forecasting was investigated by Qiu et al., who discussed the modeling mechanism and multivalue forecasting of this method, as well as its application in stock price forecasting [20]. Ince et al. discussed the NN-based time-series prediction model's establishment mechanism, then proposed adaptive time-series modeling and prediction based on a combination of the time difference method and the BPNN method and experimented with the foreign exchange rate problem as an example [21]. According to Fameliti et al., there is currently little systematic evidence that NN methods are better than traditional time-series forecasting methods in some areas. As a result, before using NN for prediction, it must be rigorously compared to traditional statistical methods to demonstrate that NN is indeed superior and then it is a wise decision to use it [22]. Shang et al. investigated the economic forecasting method, classification, and forecasting accuracy evaluation index, as well as developing a forecasting model for key macroeconomic indicators [23]. Talaat et al. proposed a new load-forecasting algorithm, the GANN optimization forecasting method [24], by combining GA and ANN. Using corporate financial data, Ding et al. developed a new discrete grey multivariate model to predict the output value of China's high-tech industries [25].

In this paper, some opinions and ideas are presented based on the predecessors' research on NN and economic forecasting models, and a high-tech industry economic forecasting model based on GANN is established. The structure and weights of GANN are used in this paper, and the NN learning algorithm is used to iterate several steps, with the network average error after iteration being used to calculate individual fitness values. Near this point, the NN learning algorithm is in charge of some quick local searches, and GA is in charge of global searches. The competition mechanism is used to adjust the structure of NN automatically. The distribution and adjustment of weights are further optimized while adjusting the structure, demonstrating more intelligence and better self-organization, self-adaptation, and self-learning ability. It not only eliminates the large approximation error caused by the fixed network model structure, but it also improves control and prediction. In this paper, GA is used to combine NN models, which overcomes the problem that NN models are prone to local minima. The simulation results show that this model's economic forecasting of the high-tech industry has a high predictive effect.

## 3. Methodology

### 3.1. High-Tech Industry and GANN Algorithm

High-tech industry refers to an industry with high knowledge and technology density, rapid industrial development, high added value and high benefit, and a certain market scale and great influence on related industries. High-tech industry is the core of all industries in the era of “knowledge economy”, the most important motive force for a country or region to gain long-term competitive advantage, and the driver of sustainable development of social economy. High-tech industry is the crystallization of the highest scientific wisdom and technology of mankind, reflecting the highest level of human production activities and management [26]. At present, the high-tech industry is developing well, and it has become an important force for mode change, structure adjustment, and steady growth. There are usually two ways to develop high-tech industries: first, based on high-tech, develop the high-tech products and form new high-tech industries. The second is to transform traditional industries with high technology. At present, we should give full play to the leading role of high-tech industries in stabilizing growth and restructuring and promote the rapid and healthy development of high-tech industries. Therefore, scientific and accurate prediction of high-tech industries is of great practical significance for government departments to grasp the future economic operation and formulate development strategies.

With the development of high-tech industries and the constant changes of society, how to establish a scientific economic forecasting system for high-tech industries, improve forecasting efficiency and accurately predict the possible development trend of industrial economy in the future has become the main goal of current research [27]. As a nonlinear calculation model, ANN expands the concept and approach of “calculating” the possibility of nonlinear systems. Its basic principle of simulating human brain and its high dimension, distributed storage and processing of neurons, self-organization, self-adaptation, and learning ability are especially suitable for economic information processing in complex modern social and economic systems [28]. The basic structural unit of ANN is neuron, which is also called node. It simulates the structure and function of biological neuron. According to the different connection modes of neurons, NN with various structures can be formed. ANN has four basic characteristics: (1) Parallel distributed processing, (2) learn through training, (3) nonlinear mapping, and (4) adaptation and integration.

**Figure 1:**
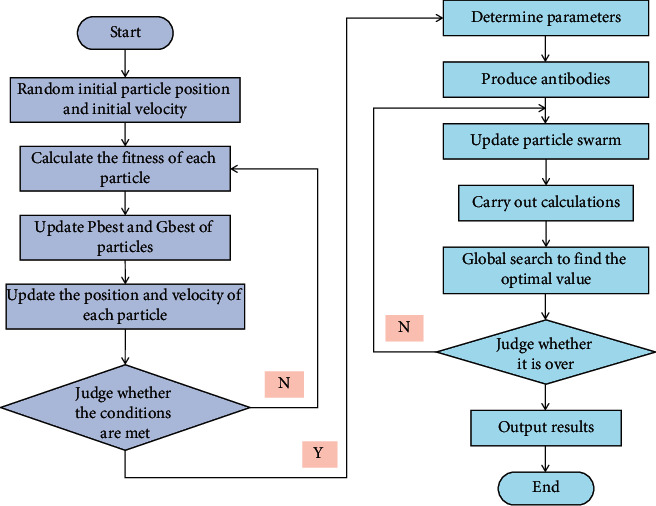
GANN algorithm flow.

Economic forecasting is essentially the forecasting of economic time series, which is a collection of unique data. The previous number will have an influence on the latter number in this set of data, and this influence can be expressed as a certain trend change or periodic change, for example. The influence relationship is usually nonlinear, and establishing a quantitative and fixed mathematical relationship is difficult. ANN is a nonlinear science. It has parallel processing, fault tolerance, and self-learning capabilities, which distinguishes it from traditional statistical methods and allows it to effectively solve such problems. This paper introduces GA as a solution to NN's shortcomings. GA refers to a collection of random search heuristic parallel algorithms [29]. The GA algorithm selects several search points at random and then conducts parallel searches from each of these search points, with the goal of obtaining points with higher-objective function values. Only the fitness guides and executes the search process repeatedly. The search points have high fitness and are close to the optimal solution after many times or generations of evolution. In NN, feed-forward NN is a common hierarchical structure. NN with various functional characteristics can be formed using various neuron transfer functions, hidden layers, and weight adjustment rules. An input layer, an output layer, and a hidden layer between the input and output layers comprise the BPNN [30]. Only one hidden layer exists in the most common NN. The goal of applying GA to BPNN is to improve learning accuracy and iteration speed, as well as to better match biological systems.  Figure 1 depicts the flow of the GANN algorithm.

By learning and training the samples, NN can constantly change the connection weights and topology of the network, ensuring that the network's output is close to the expected output. The forward NN algorithm has good local searching and climbing abilities, but it is easy to converge to the local minimum, so it is necessary to improve global convergence. To find a solution that can jump out of the local extremum and converge to the global optimal solution, the GA crossover operator and mutation operator are used. In this paper, we combine GA and NN to create an algorithm that converges quickly and finds the global optimal solution. Encoding is the process of mapping the representation of a search space solution to the representation of a genetic space solution in GA; decoding is the process of mapping the representation of a search space solution to the representation of a genetic space solution. Individuals or chromosomes are the solutions of genetic space, and multiple individuals form a group. In GA, there are several key parameters to consider, including population capacity, replacement ratio, and crossover operator. We must adjust the parameter values for different problems because the optimal values of these parameters vary from problem to problem and cannot be obtained using fixed rules.

### 3.2. Construction of Economic Forecast Model of High-Tech Industry

**Figure 2:**
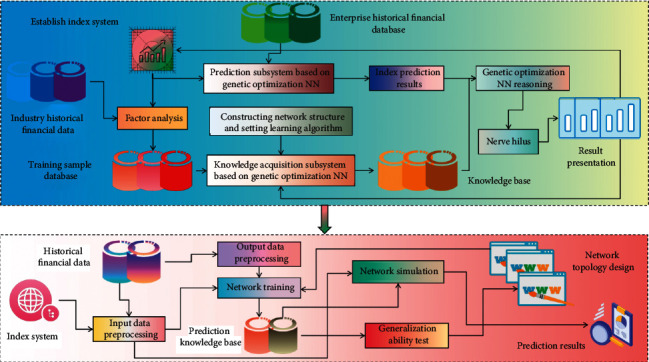
The composition of the GANN-based economic forecasting system and its subsystems.

The system applied in this paper is nonlinear, and the selection of initial weights follows the following principles: take random values and ensure that the weights are relatively small, and the output value of each neuron after initial weighting is close to zero, which can ensure that the weights of each neuron can be adjusted where their S-shaped activation function changes the most. The system is time-varying and time-delayed, so each data sample should have a different weight. For economic forecasting, the data close to the year to be forecasted should have a greater weight. In the concrete implementation, GA is used to learn the weights and offsets between layers of feed-forward network. Arrange the weights and offset values in order and generate chromosomes according to certain coding rules. In addition to the change of the connection coefficient with other neurons, the number of neurons in the middle layer can also be randomly adjusted within a certain range according to the specific situation. Once the prediction NN is preliminarily determined, its input layer and output layer will not change. GANN-based economic forecasting system and its subsystems are shown in Figure 2.

The learning rate adaptive gradient-descent method with additional momentum term is used to train the network. For each NN and its training data, there is a suitable learning rate. However, different learning rates may be required in different parts of the error surface for complex network models. When there is a large prediction error and the network needs to be corrected, the middle hidden layer is the first thing to adjust and change. A sufficient data supply is required for the establishment of multilayer forward NN. The data are primarily used to train the network and determine weights, but it is also used to check and verify the network's correctness. As a result, the information provided must be divided into two sections: training and experiment. After the decoded weights and offset values of chromosomes are substituted into the network, the fitness function is defined as the difference between the actual output and the expected output. Through GA, a new generation of people will be created on a regular basis, while those who are not fit will be weeded out. The inheritance is terminated if a specific termination condition is met. The weight and offset corresponding to the chromosome with the best fitness are currently the network's optimal solution.

Let the number of neurons in the input layer be *n*, the output layer be *m*, and the hidden layer be *p*. Then the output formula of the hidden layer is as follows:

(1) $$\begin{array}{c} x_{i}^{1}=f\left(\sum_{j=1}^{n}w_{ij}^{0}x_{j}+w_{i0}^{0}\right),\;i=1,2,\ldots ,p. \end{array}$$

Among them, *x*_*i*_^1^ is the output of neurons in the hidden layer, and ∑_*j*=1_^*n*^*w*_*ij*_^0^*x*_*j*_+*w*_*i*0_^0^ is the weighted sum of all neurons in the input layer. *w*_*ij*_^0^ is the weight coefficient of input neuron *j* to *i*. *w*_*i*0_^0^ is the threshold of neuronal *i*. *f* is a nonlinear excitation function. The calculation formula of the neuron output value of the output layer is as follows:

(2) $$\begin{array}{c} y^{k}=f\left(\sum_{j=1}^{p}w_{jk}^{1}x_{j}^{1}+w_{k0}^{0}\right),\;k=1,2,\ldots ,m. \end{array}$$

In addition, the output layer neuron error function is as follows:

(3) $$\begin{array}{c} E=\frac{1}{2}\sum_{k}\left(d^{k}-y^{k}\right). \end{array}$$

Among them, *d*^*k*^ is the target value. After calculating the error value of each node by layer-by-layer back-propagation error, the error is corrected. The weighted correction formula is as follows:

(4) $$\begin{array}{c} \Delta w_{ij}^{m}=\eta \delta_{j}^{m}y_{j}^{m-1}. \end{array}$$

Among them, *w*_*ij*_^*m*^ is the weight coefficient; *y*_*j*_^*m*−1^ is the output value of the neuron, *η* is the learning rate, and *δ*_*j*_^*m*^ is the error signal. Define the error function *E*_*p*_ as the sum of squares of errors between the expected output *d*_*pi*_ and the actual output *y*_*pi*_:

(5) $$\begin{array}{c} E_{p}=\frac{1}{2}\sum_{i=1}^{N_{L+1}}\varepsilon_{pi}^{2}\\ =\frac{1}{2}{\sum_{i=1}^{N_{L+1}}\left(d_{pi}-y_{pi}\right)}^{2}. \end{array}$$

It is hoped that *E*_*p*_ can be reduced as much as possible, so that the actual output value can be as close to the expected output value as possible. This is actually the problem of finding the minimum value of the error function, and the steepest descent algorithm can be used to make the weight coefficients change along the negative-gradient direction of the error function. The adjustment amount of the weight coefficient *W*_*ij*_^(*l*)^ can be calculated as follows:

(6) $$\begin{array}{c} \Delta_{p}W_{ij}^{\left(l\right)}=-a\frac{\partial E_{p}}{\partial W_{ij}^{\left(l\right)}},\;a>0, \end{array}$$

In the formula, *a* is the learning stride, which changes with the learning process. *l*=1,2,…, *L*;  *i*=1,2,…, *N*_*l*+1_;  *j*=1,2,…, *N*_*l*+1_.

The test data are used to determine the prediction error of the established network, and the training data are used to establish the network. The middle layer should be adjusted if the error exceeds the allowable range. When correcting the network's weight, the additional momentum method takes into account not only the effect of error on the gradient but also the influence of changing trend on the error surface. The network may fall into shallow local minima without additional momentum, but it is possible to slip through these minima with additional momentum. At the same time, making the middle layer dynamic is critical for accurate economic time-series prediction. Unlike when using NN in scientific calculations, the prediction point of NN prediction is always outside the training data, making it difficult to control the error. Furthermore, some economic fields, such as stock market fluctuations, change at a rapid pace, necessitating dynamic models to keep up.

When calculating the fitness of genetic algorithm, the reciprocal of neural network error is used to evaluate the fitness of the network, namely,

(7) $$\begin{array}{c} \text{fitness}=\frac{1}{S_{se}},\\ S_{se}=\frac{1}{2}\sum_{i=1}^{k}\sum_{j=1}^{n}{\left(d_{ij}-o_{ij}\right)}^{2}, \end{array}$$

where *d* is the standard output, *o* is the actual output, *k* is the number of input patterns contained in the training set, and *n* is the number of neurons in the output layer.

Establish an initial group. The number of individuals in the population is between 45 and 65, and the length of individuals is the product of the sum of weights and offsets of NN and the number of digits occupied by a code, and the genes of individuals are randomly selected within a predefined range. The information provided by an input neuron is not enough to establish an accurate network model to simulate complex nonlinear relationships. At the same time, the input values should show the curve characteristics of fluctuation. Therefore, in addition to expanding the one-dimensional time series, the input data should be preprocessed. On the contrary, the goal of prediction determines the single output characteristic of the network. If the total input of neurons is too far from the threshold, the modification of weights will be very small, which will not only slow the learning speed, but also make it difficult for the network to converge. At the same time, small numerical information may be overwhelmed by large numerical information. Therefore, before NN prediction, in order to avoid network paralysis caused by too large raw data, the raw data should be normalized. For the predicted value, it is not suitable to be directly used as the output of NN because of the large change range. GA algorithm is used to optimize the combination of weights and parameters of ANN model repeatedly. The goal is to set a small load error, with the hybridization rate of 0.4 and the variation rate of 0.005. According to the value range of the parameter set and the set number of individuals, the initial population is randomly generated, and the fitness value of each individual is calculated. Through systematic cluster analysis, the population is divided into several populations, and the average fitness of each population is calculated, and some individuals with the best fitness value are selected as the representatives of the population to breed the next generation.

For the same forecast project, the size of the mean square error can be used to measure the quality of different forecasting methods. For different forecast items, due to their different actual values, only comparing the size of the mean square error cannot explain the problem. At this time, *E*(|*e*|/*x*) should be used as the comparison standard, which is called the predicted mean absolute percent error (MAPE). It is estimated by retrospective forecasting, and its estimator is as follows:

(8) $$\begin{array}{c} \text{MAPE}=\frac{1}{n}\sum_{i=1}^{n}\frac{\left|e_{i}\right|}{x_{i}}. \end{array}$$

In the NN model, in order to prevent the situation that the fitting of the learned data is good and the fitting of the nonlearned data is not good, rolling learning and prediction are adopted, and the minimum sum of the error of learning and prediction is the evaluation mark of the success of the model. Generally, the quality of prediction results is measured by MAPE.  Table 1 gives the prediction accuracy range of MAPE.

Select the individual with the best fitness value in each population to continue the genetic operation until the fitness of the solution is no longer significantly increased. At this time, the decoded parameter combination is close to the best combination that meets the application needs. On this basis, the BP algorithm of ANN is used to further accurately optimize the network parameters obtained above, until the optimal network parameters are found, and then the precise optimal parameter combination can be obtained. Owing to the singularity in NN training, the results of each training will be slightly different. This also means that the prediction result of NN will be a range value, so it is necessary to evaluate the prediction result, and get the reliability and risk degree of the prediction. As GA replaces the initial optimization of NN, the network only optimizes the parameters on the basis of approaching the optimal solution, thus effectively improving the optimization speed and accuracy of the network. GA-based NN model is used in prediction. That is, the model prediction result is used as input and the actual value is used as output, and a mapping relationship is established between the model prediction result and the actual value. After continuous learning and testing, the network is applied to the economic forecast of high-tech industry to get the final forecast result.

## 4. Result Analysis and Discussion

Through theoretical analysis, the economic factors related to the GDP of high-tech industries are: labor input and capital input. Based on the GDP data of high-tech industries in a province from 2010 to 2020, this paper establishes a prediction model. MATLAB software has powerful functions of numerical calculation, data visualization, graphic drawing, and so on, which brings great convenience to users. In this paper, the NN toolbox based on MATLAB is implemented in full software. The network training involves the generalization ability of the network. The so-called generalization ability refers to the ability of NN to correctly reflect the new sample data other than the training samples. In this paper, the available samples of the training set are randomly divided into two parts: one part as the training set and the other part as the test set. Taking eight samples from 2010 to 2017 as training samples and three samples from 2017 to 2020 as test samples, the established prediction model is tested.

The fitness function is used to evaluate the superiority and inferiority of individual solutions, so as to breed or select individuals. The fitness function is not bound by continuously differentiable condition, and the domain of the function can be any set. The only requirement for the fitness function is that the input can be compared with the output. For time-series prediction, the number of input nodes can be obtained by the following methods: changing the number of input nodes from small to large, and training and testing its accuracy. When the error does not decrease further with the increase of the number of input nodes, the critical value of the change of the number of input nodes is the numerical value to be adopted. We use various forecasting methods, such as Arima model, Stepar model, and Winters model, to forecast the economy, respectively. These prediction methods are compared with this GANN prediction method, and the results are shown in Table 2.

**Figure 3:**
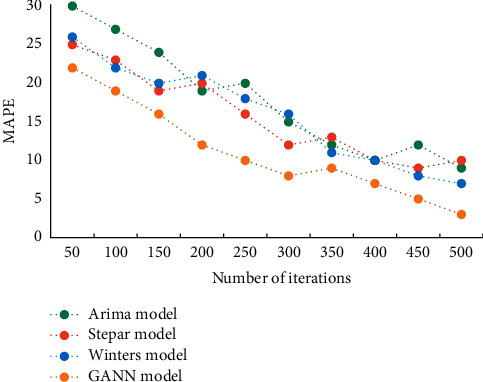
Comparison of MAPE results of different algorithms.

Draw representative MAPE results into line charts, as shown in Figure 3.

**Figure 4:**
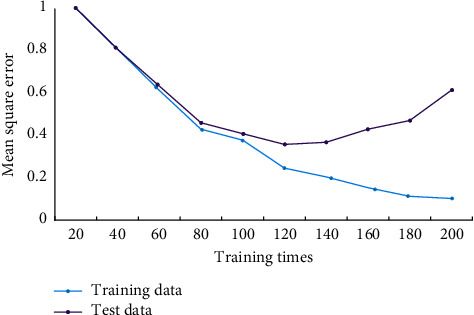
Network output error change diagram.

As a result, this algorithm outperforms the others. Dynamic NNs are those that have feedback, whether local or global. The weights and thresholds of the network can be changed, and the structure of the network, that is, the number of nodes in the input layer, hidden layer, and output layer, can be changed, so that the network is not only dynamic, but also the retained system information is more complete. After the BPNN has been generated and initialized, the network can be trained using the existing “input-output” sample vector data. The train function completes the BP network training, and the network training parameters are properly set prior to training. The train function is used to train the BP network after the training parameters have been set. The network training will alternate between training and testing, with the mean square error of each training recorded. The network weight will then be left unchanged, and the network simulation will be run forward with the test data, with the test mean square error recorded once. The curves of two kinds of mean square errors with training times can be drawn using the two types of data, as shown in Figure 4.

From the error curve, it can be seen that before a certain number of training times, with the increase of training times, the two curves decrease simultaneously. When this number of training times is exceeded, the training error continues to decrease, while the test error begins to rise. This training number is the best training number.

**Figure 5:**
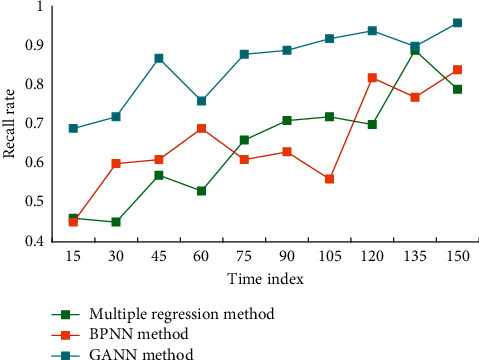
Comparison of recall rates of algorithms.

Mutation operation refers to the change between 0 and 1 in some bits when an individual generates the next generation. It plays the role of local search in GA, which increases the ability of GA to search the optimal solution. Appropriate mutation strategy can improve the diversity of individuals in the population, thus preventing GA from falling into local solution and terminating evolution. The preprocessing of input data is the key step to effectively train NN, which directly affects the performance of the trained network. In this paper, the mean and variance of the sample set data are standardized so that the mean is zero and the variance is 1. When there is new data input, we think it has the same distribution as the training sample data, so we can convert it according to the aforementioned formula. In this paper, some measures are taken to improve NN from the learning rate adaptive network learning with additional momentum term, the principle of minimizing empirical risk and the principle of minimizing structural risk, and so on, so as to make the NN model closer to the actual economic operation.  Figure 5 shows the comparison result of recall rate of the algorithm.

**Figure 6:**
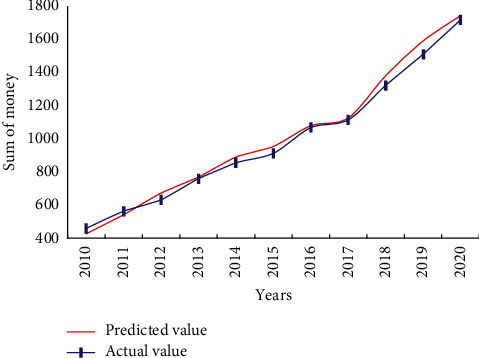
Comparison of predicted and actual values.

It can be seen from Figure 5 that the recall rate of the algorithm in this paper is higher than that of the other two algorithms, and the recall rate of the algorithm in this paper is better. Research and analyze the factors that affect the industrial technical and economic indicators, and determine some important factors as the input elements of the network. Analyze and determine some main technical and economic indicators of the enterprise as the output elements of the network, that is, the indicators that need to be predicted. Taking the influencing factors of the industry to be predicted as the input elements of the grid, input them into the model for pattern matching inference, and finally output the expected predicted value of technical and economic indicators. According to various typical rules for modifying network weights and the network training process, various network design and training subroutines are written in MATLAB language. We can call the NN design and training in the toolbox according to our own needs. Program, so that you can free yourself from tedious programming and concentrate on thinking and solving problems, thereby improving efficiency and the quality of problem solving. We compare the economic forecast results using our model with the actual values, and the results are shown in Figure 6.

**Figure 7:**
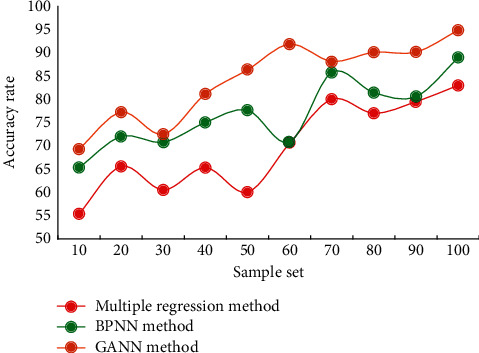
Economic forecast results of different models.

In order to ensure the generalization ability of the network, the error accuracy cannot be improved only by the number of training, and the test of the test data error is appropriately added in the training process, which can stop the network training in advance and prevent over-training. Although this method sometimes cannot achieve the preset error accuracy, it ensures the good generalization ability of the network, thus making the prediction more effective. Owing to the increasing trend of economic system index data year by year, it is easy to fall into the blind spot of prediction using historical data values to train network models and applying the trained models for prediction. Therefore, we introduce an economic indicator growth rate input node in the network input unit to improve the generalization ability of the NN. As long as the input data of the validation sample set is predicted, it can ensure that the nerve does not fall into the training blind spot. The multiple regression method, BPNN method, and GANN method are used to compare the economic forecasts of high-tech industries. The results are shown in Figure 7.

The comparative analysis of the prediction results shows that the prediction accuracy of the multiple regression method is low, and the highest is only 83.21%. The prediction accuracy of BPNN method is better, the highest is 89.17%. The GANN method proposed in this paper has the best prediction accuracy, up to 95.14%. This result further verifies the feasibility and practicability of the method in this paper. From the comparison of system modeling and prediction results, it can be seen that after using GANN, expanding the sample set, and introducing time-series data input units, the NN model meets the requirements of economic system modeling and prediction. The generalization ability of the GANN constructed in this paper has been greatly enhanced, and the system time-varying delay problem has been solved. The experimental results show that the prediction accuracy of the model in this paper reaches 95.14%, and a satisfactory prediction effect is achieved.

## 5. Conclusion

At present, the development of high-tech industry has become an important indicator to measure the comprehensive national strength, economic strength, and scientific and technological strength of a country or region. Therefore, scientific and accurate prediction of high-tech industries is of great practical significance for government departments to grasp the future economic operation and formulate development strategies. This paper adopts NN modeling method to study the establishment of economic forecasting model of high-tech industry, introduces GA to optimize NN and gives specific implementation steps. The construction scheme of this paper fully reflects the advantages of combining GA and NN technology in economic forecasting and has strong normative and operability. In order to verify the performance of the model, through simulations, predictions are made for the instances. The results show that the algorithm in this paper has faster convergence speed and greater generalization ability, and the average error rate is reduced to about 1%. The algorithm improves the learning and prediction accuracy of NN, and its accuracy reaches 95.14%. At the same time, the good flexibility makes it well suited to many economic forecasting fields. The research of this paper provides a certain basis for the economic forecast of the high-tech industry and the government's regulation of economic growth and has certain practical significance. The construction idea of this paper has certain reference value for most enterprises to construct their own economic forecasting system. However, due to the limitation of knowledge level, time and energy, there are still some problems in this paper. For example, the entire economic forecasting system is based on the authenticity of the data. If the original data are not true, the final forecast result will not have a reference. Value; since the network is dynamic, the number of neuron nodes and the number of hidden layers are adjusted during the training process, which also makes it difficult to prove the stability of the network. These problems need to be expanded and further researched to solve.

## Figures and Tables

**Table 1:** Prediction evaluation based on MAPE.

Scope of MAPE	Prediction and evaluation
MAPE ≤ 8%	High precision prediction
8% ≤ MAPE ≤ 25%	Good prediction
25% ≤ MAPE ≤ 55%	Feasible prediction
MAPE ≥ 55%	Misprediction

**Table 2:** Comparison of different methods.

Method	Square sum error	Mean absolute error	Mean square deviation	Average percentage error	Mean square percentage error
Arima model	2216.8	4.59	0.54	3.11	0.53
Stepar model	2119.6	3.78	0.29	3.63	0.55
Winters model	1987.2	3.96	0.37	2.26	0.42
GANN model	214.9	2.13	0.21	1.01	0.31

## Data Availability

The data used to support the findings of this study are available from the corresponding author upon request.
